# Oral Immunotherapy for Children with Cow’s Milk Allergy

**DOI:** 10.3390/pathogens10101328

**Published:** 2021-10-15

**Authors:** Mika Ogata, Jun Kido, Kimitoshi Nakamura

**Affiliations:** 1 Department of Pediatrics, Faculty of Life Sciences, Kumamoto University, Kumamoto City 860-8556, Japan; ogata.mika.dy@mail.hosp.go.jp (M.O.); nakamura@kumamoto-u.ac.jp (K.N.); 2 Department of Pediatrics, Graduate School of Medical Sciences, Kumamoto University, Kumamoto City 860-8556, Japan; 3 Kumamoto Medical Center, Department of Pediatrics, National Hospital Organization, Kumamoto City 860-8556, Japan

**Keywords:** baked milk, cow’s milk allergy, oral immunotherapy, tolerance

## Abstract

Cow’s milk allergy (CMA) is one of the most common IgE-dependent food allergies in children. Some children develop severe and persistent CMA, with near-fatal reactions after exposure to trace amounts of cow’s milk (CM). Because milk and dairy products are included in various processed food products, it is difficult to completely remove milk, which negatively affects the quality of life of children with CMA. Oral immunotherapy (OIT) can alleviate food allergen-induced anaphylaxis under continuous ingestion of a little of the causative food. Children with severe CMA may benefit from OIT, but the treatment requires a long time and poses a risk of anaphylaxis. Moreover, in recent years, new therapies, including omalizumab, sublingual immunotherapy, and epicutaneous immunotherapy, have played the role of optional OIT. In this review, we present the current methods of and other attempts at OIT, and discuss OIT for safely treating CMA.

## 1. Introduction

Cow’s milk allergy (CMA) is one of the most common IgE-dependent food allergies in children, affecting 0.5–3% of children [1,2,3,4,5,6]. CMA is developed in the first year of life and is likely to be outgrown with age [2,7,8,9,10]. The outgrowth rates may be lower in high-risk cases of CMA, such as those with high levels of cow milk-specific IgE [9].

Cow’s milk (CM) contains approximately 30–35 g of proteins per liter [11], comprising more than 40 different proteins. In CM, casein and whey account for 80% and 20% of milk proteins, respectively. The major allergens of CMA are caseins (Bos d 8), α-lactalbumin (Bos d 4), and β-lactoglobulin (Bos d 5) in whey (Table 1) [12]. Most children with CMA are polysensitized to these proteins [13,14].

The basic aspect of food allergy management is the avoidance of causative foods. CM and its products are major sources of protein and calcium in the diet of infants and young children. Bone mineral density is likely to be low in CMA children, which may persist into adulthood [15] and prepuberty [16], because a milk-free diet may negatively affect bone development in growing children. Moreover, adolescents with CMA from infancy are at risk of not achieving their expected height and bone growth [17,18,19].

Some children develop severe and persistent CMA, with near-fatal reactions after exposure to trace amounts of CM. Because milk and dairy products are included in various processed food products, it is difficult to completely remove milk and dairy products from the diet, which negatively affects the quality of life of children with CMA.

A Canadian survey that performed a questionnaire study with parents of children with multiple food allergies, such as CMA and hen’s egg allergy, reported that CM was the allergen food with the greatest time, financial, social, and emotional burdens compared to foods such as hen’s eggs and peanuts [20]. Parents expected that medical treatments could permanently cure their children’s food allergies and reduce their fear of severe allergic reactions if a cure was difficult [21].

Oral immunotherapy (OIT) is currently a curative treatment option for food allergies. The European Academy of Allergy and Clinical Immunology (EAACI) states that OIT may increase the amount of food that children can tolerate, alleviate allergic symptoms, and reduce the risk of potentially life-threatening allergic reactions [22]. Palforzia, a peanut OIT product, was approved by the United States Food and Drug Administration (FDA) in 2020 to treat children with a peanut allergy [23]. The EAACI guidelines indicate that oral immunotherapy for CMA is a curative option to increase the threshold of allergy reaction in children with persistent CMA while on the treatment [22].

Herein, we explain OIT for children with IgE-mediated CMA in Japan and overseas, evaluate the methods of OIT for CMA, and analyze the effect of OIT for CMA. Moreover, we discuss the future OIT methods for CMA.

## 2. OIT for CMA

CM is one of the most common causative foods for food-induced anaphylaxis, and is a significant causative food for severe reactions [24,25]. We summarized the previous results of OIT for CMA through the literature review (Table 2). OIT for CMA can alleviate CM or dairy product-induced anaphylaxis through the ingestion of a bit of the allergen derived from CM. However, this therapy is associated with severe adverse reactions, with anaphylaxis occurring in some cases [26]. In previous reports, 15–20% of children with CMA had to discontinue treatment due to the significant side effects [27,28,29,30]. Moreover, the therapeutic effect of OIT for CMA does not usually persist once treatment is discontinued. In a follow-up study by Keet et al., less than one-third of children with CMA were asymptomatic and could outgrow it at three to five years after undergoing milk OIT [31].

## 3. Indication of patients undergoing OIT for CM

Several guidelines and consensus regarding OIT for IgE-mediated food allergies have been published in recent years [22,52,53,54,55], but standardized protocols have not been established, except for peanut OIT.

OIT is potentially indicated for infants or children with evidence of an IgE-mediated CMA. For infants or children with CMA, avoidance therapy may be ineffective, undesirable, or even cause severe limitations to their quality of life (QOL). A definite diagnosis of CMA is essential before proceeding with OIT. If infants or children do not have a clear medical history of immediate reactions after intaking CM or dairy products, they need an oral CM or dairy product challenge test [22,52,54]. The baseline reaction threshold should be used to establish the efficacy of OIT in individuals [22]. Individuals with non-IgE-dependent gastrointestinal allergies or lactose intolerance are not eligible and must be excluded. Because of the burden, such as the long-term treatment and common adverse reactions, that is placed on patients and their families, they should be motivated and adherent, and may have to take emergency medical treatment in the case of adverse effects.

Treatment adherence is essential, because it might result in a higher rate of allergic reactions. Uncontrolled asthma and severe atopic dermatitis must be controlled before starting OIT. Individuals with mastocytosis, malignant neoplasms, systemic autoimmune disorders, pregnancy, and disorders or treatments with contraindications to intramuscular adrenaline may not be considered as OIT candidates [22,52,54]. Histories of anaphylactic reactions to the targeted food allergen are generally not an exclusion criterion in OIT studies [22,52,54].

## 4. Protocols of CM-OIT

Standardized protocols in OIT for patients with CMA have not been definitely established. The optimal form of milk protein to be ingested (liquid, powdered, or baked milk, dairy products, etc.), start and target doses, and duration of treatment have not been established [22,52,54].

**Figure 1:**
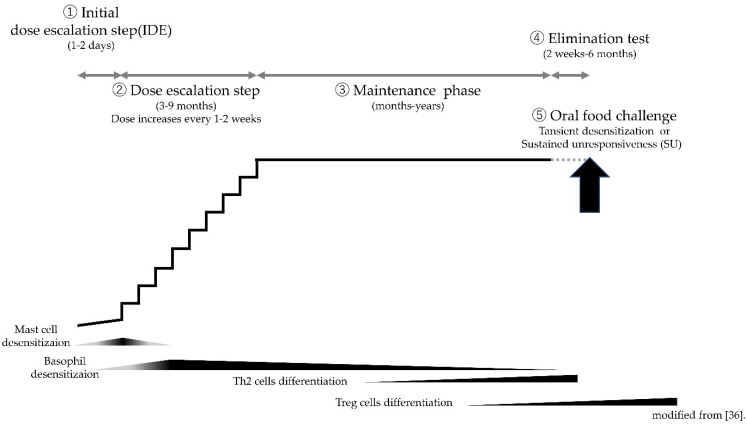
Standard OIT protocol for children with CMA.

In OIT for individuals with CMA, increasing amounts of milk protein during regular intervals is essential for reducing the sensitivity to CM allergens and preventing the development of clinical manifestations. Typical OIT protocols consist of three steps; namely, initial dose escalation (IDE), dose escalation, and maintenance (Figure 1) [56]. Some protocols utilize a clustering protocol with a fixed dose in the first one to two days during IDE. Others settle on an individualized amount on the basis of the obtained results in the baseline OFC. In the former protocols, increased doses are ingested every 30–60 min. In the latter rush-desensitization protocol, the initial dose is started in 1/10 to 1/2 of the threshold (the setting varies depending on the study). The amount is increased by 20–100% every several hours, and the maximal ingested amounts are completed in three to seven days [52,56]. Many studies conducted in Japan utilize this rush protocol. The tolerated dose during OIT determines the start dose in the dose escalation phase. In the dose escalation period, patients take the OIT dose under medical supervision and repeat it daily at home for one to two weeks. The dose of OIT may be increased on the basis of the protocol during healthcare until the targeted maintenance dose is achieved or until adverse symptoms appear. The maintenance doses are given for months to years, with the ultimate goal being to attain permanent tolerance. There is no evidence of the required minimal duration of the maintenance phase [56].

In OIT, adverse reactions such as anaphylaxis may frequently develop in many cases [26,57]. Infants or children with CMA and their caregivers must recognize and treat these adverse reactions as soon as possible. Moreover, it is necessary to prepare emergency care in case of adverse events. Clinicians should prescribe self-injected adrenaline and provide medical information to a nearby emergency hospital to safely deliver this therapy.

## 5. OIT Issues

### 5.1. Is It More Effective to Start Treatment at a Young Age?

Approximately 50–90% of CMA subjects are expected to outgrow their allergy by the time they are five years old [7,8,9,10,32]. Most guidelines for OIT recommend induction for children with persistent CMA when they are age of four to five years [22]. CMA children with high blood milk-specific IgE levels [8,9,10,32,58] are less likely to achieve spontaneous outgrowth. Moreover, because these patients may develop severe reactions to even small amounts of CM proteins, early intervention may effectively prevent severe reactions after accidental exposure to CM [32,52]. The majority of previous studies have been performed for severe CMA cases. Cases with higher CM-specific IgE were likely to be at higher risk for allergic reactions to CM-based OIT [33]. Earlier OIT might allow for less sensitized infants to tolerate CM, and might identify more severe ones to avoid fatal food reactions [34,35].

Martorell et al. performed OIT for two-year-old (24–36-month-old) children with CMA [36]. Of the children in the OIT group, 90% (*n* = 30) were able to ingest 200 mL of CM without adverse reactions, while the outgrowth rate in the control group (*n* = 30), who continued a milk-free diet, was only 23%. The outgrowth rate in the OIT group was higher than that in the spontaneous tolerance group. However, 80% of the OIT group (*n* = 30) showed some allergic reactions, and one patient needed adrenaline, but the control group was also comparable.

In OIT studies on infants of less than one year old [34,35], 97–98% of the subjects have been shown to ingest CM without severe allergic reactions, with a few experiencing severe allergic reactions.

The baseline CM-specific IgE level, the wheal size in the skin-prick test (SPT), and the severity of atopic dermatitis in individuals are likely important outgrowth predictors for CMA [10,59]. Other predictors of persistent CMA are a higher peak level of CM- or casein-specific IgE [8,9,58], complications of asthma or allergic rhinitis [9], and a history of anaphylaxis [60]; cases with these factors are at a high risk of adverse reactions to OIT [33,37,61]. This OIT has been performed for infants [34,35,36], including those with low CM-specific IgE or spontaneous tolerance. Therefore, in terms of efficacy and safety, we cannot simply compare the results of OIT for infants to those for older CMA children with higher specific IgE. Early intervention in subjects with persistent and severe CMA is expected to be effective.

### 5.2. Role of An Initial Dose Escalation Phase

OIT protocols are generally started with an initial dose escalation phase, which involves ingesting a small amount of milk below the threshold and escalating the dose level over the first few days [57]. In the Palforzia^®^ treatment protocol, initial dose escalation is administered in sequential order on the first day from 0.5 to 6 mg of peanut protein [23]. If successful in IDE, children with CMA are ready to move up a dosing phase. Mast cells and basophils are considered to be essential cells with important roles in rush desensitization, particularly in IDE, because some mediators are promptly released from these cells during allergic reactions [62,63,64].

#### 5.2.1. Mast Cells

In mouse models of passive anaphylaxis, short-term desensitization, progressively increasing antigen levels, could prevent the degranulation of their mast cells [62,63,65,66]. Murine studies have shown that such repeated exposure to low levels of allergen induces endocytosis of surface-bound IgE specific to its allergen on mast cells [65] and inhibits Ca^2+^ flux by actin remodeling in mast cells [66], resulting in their suppression. Because of the difficulty in sampling tissue-resident mast cells, there are insufficient data on human mast cells in OIT [62,63]. Therefore, further research is needed to evaluate their role in OIT.

#### 5.2.2. Basophils

Basophils, similar to mast cells, are essential effector cells in IgE-mediated food allergies [67]. The basophil activation test (BAT), measured by the upregulation of CD63 and/or CD203c, is an in vitro functional test resembling an in vivo oral provocation test. BAT is used to identify the culprit allergen and, more recently, to monitor the clinical response to OIT [27,67,68,69,70,71]. Allergen-induced basophil hyporesponsiveness occurs within the first few months of OIT, but this initial suppression appears to be temporary. Though continuous exposure to a low-dose allergen may induce transient clinical desensitization [62,63,64], adverse reactions become provoked in many cases once therapy is discontinued. There seems to be a correlation between basophil suppression and clinical desensitization in OIT [71,72].

Adverse reactions often occur within the first weeks or months of OIT. OIT with exercise, infection, and menses may induce allergic reactions [22,38,73]. Mast cell and basophil desensitization induced in the initiation phase of OIT seems to be a strategy to raise the threshold and tolerance to subsequent escalating antigen doses.

In the early stages of OIT, the wheal diameter of SPT decreases with a decrease in basophil reactivity [63]. Kido et al. suggested that SPT is more clinically valuable for diagnosing CMA and predicting CMA outgrowth than milk-specific IgE. They reported why SPT can reflect the amount of tissue-fixed IgE, unlike specific IgE in circulating blood [74]. The difference between SPT and serum-specific IgE in test results might reflect immune response differences in the skin and blood [75]. Because of the high affinity for IgE of FcεRI on mast cells or basophils, the IgE equilibrium is considered to be more predominant in tissue pools than peripheral blood [76,77]. In addition, SPT may link to kinetics of cytokines other than IgE. A previous study about six-year-old children with elevated specific IgE levels to house dust mites indicated that the SPT wheal diameter is positively correlated with the IL-4, IL-5, and IL-13 (Th2 cytokines) responses, and negatively with IL-10, a regulatory cytokine [78].

The fact that basophils only live a few days, whereas mast cells live for weeks to months, also suggests important questions about the contribution of each cell type to the dynamics of desensitization [63]. Understanding both mast cell and basophil kinetics, as well as the subsequent responses raised in other effector cells by these cells, is important to safely perform OIT in clinical practice.

### 5.3. Are Lower Maintenance Doses Safer?

The maintenance dose for CM-based OIT is typically a daily dose of 200 mL of CM. Takaoka et al. [39] evaluated the efficacy and safety of low (20 mL)- or high (100 mL)-maintenance target doses of OIT in children with severe CMA. There was no significant difference in the final OFC dose between the two groups, but severe symptoms in the maintenance phase were less frequent in the low-dose group. Meglio et al. desensitized children with CMA who were unable to achieve a CM maintenance dose of 200 mL with 40–80 mL of CM. This resulted in reducing the risk of severe reactions after accidental CM intake [40]. Moreover, in a similar study, 15 mL of CM, as a maintenance dose, allowed to increase the threshold and immunological changes [28]. Adverse reactions were not severe in this study. Decreasing the target dose to less than 200 mL may reduce the frequency of severe allergic symptoms caused by OIT. Yanagida et al. [41] performed OIT, aiming at 3 mL of milk for severe CMA children without tolerance of 3 mL or less of CM. At both one year after receiving OIT of CM and a two-week elimination period, 58% of the participants were able to consume 3 mL of CM. Moreover, 33% could tolerate 25 mL of CM, while the casein-specific IgE levels decreased and casein-specific IgG_4_ increased compared to the baseline in the OIT group. Adverse allergic reactions rarely occurred, and most allergic symptoms were mild even when they did develop [41]. High (3000 mg of peanut protein)- and low (300 mg)-dose peanut OIT similarly suppressed proallergic cytokines and basophil activation in young children [79]. Even a lower maintenance dose than 200 mL of CM could induce both immunological changes and an increased threshold dose above the maintenance dose. As much as 200 mL of CM may not be necessary if sustained exposure to low allergen levels can cause immunological changes. OIT can increase the threshold dose for allergic reactions and substantially reduce the risk of severe allergic reactions after accidental ingestion of the allergen [22,30,80]. Levy et al. reported that 180 mg of milk protein, the “minimal protective dose,” might prevent allergic reactions after accidental consumption, even if unable to successfully intake 200 mL of milk. They suggested that a start dose higher than 30 mg of milk protein is one of the clinical predictors for achieving a full dose [42]. Determining the start dose is important for safely performing OIT for children with CMA [81]. The eliciting dose (ED) value refers to the threshold dose predicted to elicit objective allergic symptoms. Several ED values have been proposed by different oral food challenge studies [82,83,84]. ED_10_ levels predict that 10% of individuals allergic to culprit foods present adverse reactions at this dose. The ED_05_ and ED_10_ of milk protein are 1.1–2.4 mg (0.03–0.07 mL of CM) and 4.2–7.1 mg (0.1–0.2 mL), respectively [82,83]. The ED value may be useful for subject selection and risk stratification.

CM-based OIT is a significant risk factor for epinephrine-treated reactions compared with non-CM-based OIT [85]. Dose adaptations or order-made schedules of OIT are produced according to the severity of the allergic reactions in the dose escalation phase [22]. In the maintenance phase, dose adaptations are also needed, since safety should be the priority in administering OIT.

### 5.4. Is Daily Intake Necessary?

In the maintenance phase of CM-based OIT, no difference in clinical and immunological efficacy between daily and twice weekly intake regimens has been observed [86]. Poor adherence is an absolute contraindication. OIT is time consuming and burdened by potential side effects [22]. Participants and their families must be highly reliable and committed to a treatment regimen that may cover an extended period of time [22]. Epstein-Rigbi et al. investigated the quality of life (QOL) of food-allergic children before, during, and after OIT [87]. Their QOL was temporarily exacerbated at the initiation of OIT, but improved after finishing the maintenance phase. However, daily intake is a burden on children and their guardians [86]; thus, more flexible regimens are needed considering their QOL and efficacy [22].

### 5.5. Is More Prolonged Treatment More Effective?

Although OIT desensitizes and increases the threshold in most subjects, they regain clinical responsiveness once discontinuing daily dosing [22,63,72]. There is no published evidence on the minimal duration of the maintenance phase to achieve tolerance [53]. In the first place, there are no clinical or immunological criteria to evaluate the tolerance of food allergies [22,64], and there is no evidence that OIT can cure food allergies [80]. The first aim of OIT is to increase the reactive threshold and protect food-allergic individuals from severe allergic reactions due to accidental ingestion [22,80]. SU refers to a state maintaining the clinical effect of OIT for a while after treatment cessation, which is different to true immunologic tolerance. Current studies assess the therapeutic effects of OIT by the achievement of SU, allowing for flexible consumption of the culprit food. The required duration to diagnose SU is not defined and varies from two weeks to six months [64,80]^.^

Highly predictive biomarkers for sustained unresponsiveness (SU) are needed [22,63]. Lower milk- or casein-specific IgE levels at the start of OIT are associated with achieving SU [31,43,88]. Moreover, other immunological markers are likely to relate to SU. OIT induces changes in avidity, with IgE and IgG_4_ binding to milk protein epitopes. Lower binding and diversity of specific IgE to allergenic epitopes, CM peptides, are more likely to achieve SU [88,89]. In peanut OIT, early decreases in basophil sensitivity to Ara h 2 correlate with SU [69,71]. While some cases can achieve SU, others achieve partial desensitization, but it is not known whether qualitative differences in immune mechanisms exist between them [62].

Moreover, most patients that achieve SU tend to have a smaller wheal diameter in their SPT and lower basophil activity to allergens than those that do not achieve SU [27,44,69,71,72,74]. Some studies have suggested that a more prolonged duration of OIT may increase SU [90,91]. While SU seems to differ from permanent tolerance, it is unknown whether SU is a state of simple desensitization that induces extended maintenance therapy or an intermediate state between desensitization and tolerance [63]. Thus, future studies about immunological changes associated with SU are needed.

## 6. Use of Omalizumab

The use of biologics, especially omalizumab, demonstrates promising effects in OIT [43,44,45,46,92]. Omalizumab treatment before initiating OIT facilitates rapid dose escalation [92] and decreases allergic reactions [45]. Within the first weeks or months of starting OIT, specific-IgE levels increase from the baseline when the highest rate of allergic reactions occurs [63]. Omalizumab seems to reduce serum specific-IgE levels and downregulate FcεRI levels on mast cells and basophils [93]. However, low specific IgE levels are not always necessary for the desensitization of these cells, because the desensitization of mast cells and basophils occurs even when specific IgE levels are high [63].

In a cohort study of children receiving high-dose CM-based OIT with omalizumab, the CD4^+^ T cell responses to CM were nearly eliminated within a week after receiving treatment [94]. In a similar peanut OIT study by Abdel-Gadir [95], the proliferation of allergen-specific effector T (Teff) cells and regulatory T (Treg) cells precipitously declined following the initiation of omalizumab therapy prior to OIT. These peanut-specific Treg cells showed a Th2-like phenotype at baseline and could not suppress peanut-specific Teff cells. Treg cells are reprogrammed by a Th2 cell-like phenotype in food allergies. Therefore, Th2 cell-like Tregs promote food allergies and prevent oral tolerance in OIT [96]. Omalizumab may promote allergen desensitization by depleting allergen-reactive T cells in OIT. Subsequently, improved Tregs reverse the Th2 cell-like program of Tregs and restore their function, resulting in the lessening of food allergies [95]. However, the in vitro addition of IL-2 or anti-IL-10 fails to rescue the decreased proliferation of peanut-specific CD4^+^ Teff or Treg cells. This suggest that anergy is not a major mechanism for the decline in peanut reactivity. Moreover, suppression via omalizumab may not be associated with anergy or IL-10-mediated suppression [63,95].

Omalizumab, which plays the role of adjuvant in OIT [97], reduces adverse effects; however, it does not affect desensitization or SU [43]. Most cases lose the protective effect after discontinuation of omalizumab [45,46,97]. Recently, clinical trials with dupilumab were initiated for milk [98] and peanut [99] OIT. New biomarkers that can identify those subjects likely to benefit from such biologics are needed [100].

## 7. Sublingual Immunotherapy (SLIT) or Epicutaneous Immunotherapy (EPIT)

SLIT is a treatment that reduces allergen syndromes by providing patients small doses of the substance causing their allergy under their tongue over an extended period of time. Meanwhile, EPIT is a potential alternative to OIT and SLIT. EPIT provides cutaneous exposure to microgram quantities of food allergens [57]. SLIT or EPIT may be a treatment option, but neither will be as effective as OIT [101] if the food-allergic subjects have difficulty performing OIT due to their low threshold.

SLIT has been evaluated in some clinical trials [27,102,103]. Keet et al. evaluated the efficacy of SLIT alone or SLIT followed by OIT to treat milk allergy. OIT was more effective for desensitization to CM than SLIT alone was, but was accompanied by more systemic side effects [27]. For peanut allergy, 32 of 48 participants tolerated 750 mg of peanut protein after three to five years of SLIT. In addition, 12 of them were able to consume 5000 mg of peanut protein. Peanut SPT, specific IgE levels, and basophil activation also decreased after treatment, and specific IgG_4_ levels increased [102].

There are many clinical trials of EPIT, especially for peanut allergy. EPIT for children with peanut allergy is safe, well-tolerated, and compliant. However, according to a lack of treatment efficacy, EPIT is still in the research phase [104]. In a pilot study, EPIT for milk-induced eosinophilic esophagitis improved their symptoms [105]. EPIT may also be useful for IgE-dependent milk allergy [106,107].

## 8. Approaches Other Than Standard OIT

Of milk-allergic children, 75–80% can tolerate baked milk (BM) [47,108]. The ability to tolerate BM can be a marker predicting mild and transient milk allergy [109]. Children able to take BM were shown to have reduced basophil activity [110], diversity of IgE epitopes, and avidity [111], as well as higher milk allergen-specific Treg cells [112]. In addition, they were more tolerant to unheated milk [48,49,109]. CMA cases who tolerate BM have better prognosis and are more likely to attain tolerance [113]. However, even BM causes anaphylaxis in some cases [49,50].

**Figure 2:**
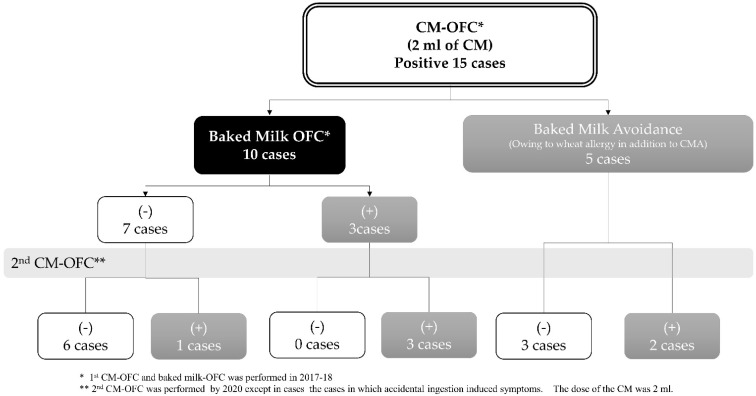
Oral baked milk challenge test in our institution. * The first CM-based OFC and baked milk-based OFC was performed in 2017–2018. ** Five patients with positive responses in the CM-based OFC avoided the BM-based OFC owing to presenting wheat allergy. The second CM-based OFC was performed in 2020, except in cases in which accidental ingestion induced symptoms. The CM dose was 2 mL.

Our institution investigated the effect of BM in 15 children with CMA who had developed allergic symptoms in the digestion of less than 2 mL of CM-based OFC (Figure 2). Next, we performed BM-based OFC in ten CMA children without a wheat allergy using a slice of bread (60 g) containing CM protein equivalent to 5–7 mL of milk. Seven children with CMA were BM-tolerant in the BM-based OFC, and six of them could consume 2 mL of CM one year after receiving BM-based OIT. In contrast, three subjects who were allergic to BM required the sustained complete elimination of CM protein. This investigation suggested that some cases who were allergic even to small amounts of CM might be able to consume BM. However, cases with allergic symptoms, even to BM, might not resolve CMA compared to those with BM tolerance (*p* = 0.033).

On the other hand, five children with CMA could not consume bread owing to presenting wheat allergy, and were treated with complete elimination of CM proteins, including BM. In addition, 60% (3/5) were able to consume 2 mL of milk one year after the elimination of CM proteins, and their clinical prognosis was similar to that of the BM group. Therefore, this investigation suggested that BM consumption might not necessarily contribute to CMA remission, although more case studies are needed.

Partially hydrolyzed milk (pHF) is used in Japan for healthy infants with or without a family history of allergy [51]. Its molecular weight is less than 5000 Da, unlike highly hydrolyzed milk with a molecular weight of less than 3500 Da. Since pHF is not approved as a hypoallergic formula [114], it is not available for children with CMA [115]. Kido et al. reported that 75% of CMA children can tolerate pHF [116]. Moreover, they evaluated SPT using pHF for predicting CMA outgrowth [74]. pHF is less allergenic than regular milk formulas are, but retains a small amount of milk protein [117]. They expected that the early and long-term administration of pHF would induce immunologic tolerance to CM antigens in children with CMA [116]. Moreover, Inuo et al. performed OIT using pHF for CMA children aged one to nine years. They demonstrated that 63% of cases with confirmed CMA could drink 20 mL of pHF with no allergic symptoms [118]. pHF intake increased the threshold after treatment, while extensively hydrolyzed milk (eHF) intake did not show this phenomenon [51]. No severe reactions appeared during the treatment, indicating its potential as a candidate as a safer treatment strategy for CM-based OIT.

## 9. Future Prospects in OIT for CMA

Standardized protocols for OIT for CMA have not yet been established. Thresholds and treatment responses may differ among different subjects. We need objective indicators to estimate the appropriate dose and duration of therapy to practice personalized treatment. For subjects with extremely low thresholds, OIT combination therapy after pretreatment with SLIT or EPIT using hypoallergenic milk proteins such as baked milk or pHF may be effective to raise the threshold, thereby promising safer OIT.

The form of dairy products used in OIT for CMA should also be discussed. Since it is difficult to precisely measure small amounts of milk protein at home, standardized and quantified allergen preparations, such as Palforzia used for peanut allergy, should be developed. Subjects with difficulty in reaching maintenance doses can be treated with a combination therapy of biologics such as omalizumab and OIT. Early intervention from infancy may improve the long-term prognosis. The prophylaxis of peanut [119,120] and egg [121] allergies is recommended to begin in infancy. Even in infants who have already suffered CMA, active therapeutic intervention from infancy may be effective. Therefore, in the future, new order-made OIT methods that include combination therapy of standard OIT methods and new treatment options, including SLIT or EPIT, hypoallergenic milk proteins, omalizumab, and other biologics, are expected to be performed from infancy, depending on the severity or ability of each subject.

## 10. Conclusions

Children with CMA persistently face a heavy burden in their diet and social life from infancy. Although many children with CMA achieve spontaneous remission of CMA, many clinicians have difficulty in determining which case to treat and when they should be treated, considering the risks and benefits of CM-based OIT. Prolonged and severe cases with CMA may be eligible for OIT. Therefore, we need an objective indicator for both the prognosis and severity of CMA. Although there is no standardized treatment protocol, the initial and maintenance doses, and the need for omalizumab as an adjuvant therapy, may vary depending on the immune characteristics of the subjects. Understanding the dynamics of basophils and Teff cells, associated with remission or SU, may contribute to achieving the goal of therapy. In order to establish safer and more effective treatment protocols, clinicians and basic science researchers should collaborate to elucidate the immunological character of patients and changes caused by treatment.

## Figures and Tables

**Table 1:** Allergens in cow’s milk.

Protein Name	Allergen Name	Molecular Mass (kDa)	Allergenicity	Others
**Curd—Casein family: 80%**				
Caseins	Bos d 8	20–30	Major	Main protein fraction of cow’s milk consists of αs1-, αs2-, β-, and κ-caseins
αs1-casein	Bos d 9	23.6	Major	Main fraction of casein
αs2-casein	Bos d 10	25.2	Major	
β-casein	Bos d 11	24	Major	
κ-casein	Bos d 12	19	Major	
**Whey (lactoserum): 20%**				Loses IgE binding following 15–20 min of boiling at >90 °C
α-lactalbumin	Bos d 4	14.2	Major	Present in the milk of almost all mammals
β-lactoglobulin; protein family: Lipocalins	Bos d 5	18.3	Major	−65% of all whey proteins absent in human milk
Bovine serum albumin; family: Serum albumins	Bos d 6	67	Minor	Clinical cross-reactivity to raw beef
Immunoglobulins; family: Immunoglobulins	Bos d 7	160	Minor	Mostly IgG
Lactoferrin; family: Transferrins		800	Minor	Loses IgE binding following 15–20 min of boiling at >90 °C

Based on the EAACI Molecular Allergology User‘s Guide, Matricardi et al., *Pediatric Allergy and Immunology*, 2016 [12].

**Table 2:** Literature review of CM-based OIT studies.

Author (Year)	*n*	Age (Years)	Maintenance Dose of CM (mL)	Anaphylaxis during OIT	Target Dose of CM (mL)	Complete Desensitization	Others
Keet (2012) [27]	30	6–17	0.2 mL (SLIT) and 30 or 60 mL (OIT)	Adrenalin was used for two SLIT doses and four OIT doses	240	10% (SLIT) and 70% (OIT)	SLIT
Skripak (2008) [28]	13	6–17	15	Four subjects used adrenalin	100	30.8%	
Staden (2007) [30]	25	0.6–12.9	100	8% of subjects experienced wheezing	150	48%	
Longo (2008) [32]	30	5–17	150	Four subjects used adrenalin injector in the hospital, and one used it at home	150	36%	
De Schryver (2019) [33]	41	6–18	200	15.8% (two cases experienced severe anaphylaxis)	200	73.2%	
Berti (2019) [34]	73	0.25–0.9	150	No infants needed an adrenalin injection	150	97%	OIT for infants
Boné Calvo (2021) [35]	335	<1	150–200 (infant formulae)	1.3%	150–200 (infant formulae)	98%	OIT for infants
Martorell (2011) [36]	30	2–3	200	One subject used an adrenalin injection	200	90%	OIT for infants
Vázquez-Ortiz (2013) [37]	81	5–18	200	Nine children were administered an adrenalin injection	200	71.6%	
Narisety (2009) [38]	15	6–16	3–480	Four subjects used an adrenalin injection	480	33%	
Takaoka (2020) [39]	33	9 (median)	20 (low-dose group) or 100 (high-dose group)	0.1% (low-dose group) and 0.5% (high-dose group)	20 or 100	90% (20 mL of CM) 34% (100 mL of CM)	Low-dose OIT
Meglio (2004) [40]	21	6–10	200	Two subjects experienced moderate asthma	200	71.4%	
Yanagida (2015) [41]	12	>5	3	A severe reaction occurred in one of 3795 doses	3	75.0%	Low-dose OIT
Levy (2014) [42]	280	3–27	240	45.7% (induction phase) and 15.7% (home dosing)	240	62%	
Wood (2016) [43]	28	11.7	300	One participant used an adrenalin injection	180	88.9%	Omalizumab
Nadeau (2011) [44]	11	7–17	60	Three subjects used an adrenalin injection	60	81.8%	Omalizumab
Martorell (2016) [45]	5	3–11	200	Three subjects experienced anaphylaxis after discontinuing omalizumab	200	100.0%	Omalizumab
Ibáñez-Sandín (2021) [46]	58	6.3–13.2	180	36.4% of subjects who discontinued omalizumab experienced anaphylaxis	180	83.0%	Omalizumab
Nowak-Wegrzyn (2008) [47]	100	2.1–17.3	1.3 g of baked CM protein	None	240	9%	Baked milk
Esmaeilzadeh (2018) [48]	42	0.5–3	First, 1.3 g of baked CM protein, and then 4.6 g of it	No data	240	88.1%	Baked milk
Gruzelle (2020) [49]	64	2–16	168.6 mg of baked CM protein	Six subjects experienced asthma (one subject used two injections of adrenalin)	254	42.2%	Baked milk
Goldberg (2015) [50]	15	6–12	1.3 g of baked CM protein	Three subjects used an adrenalin injection	1.3 g of baked milk protein	21%	Baked milk
Inuo (2018) [51]	25	1–9	20 mL of pHF	None	20 mL of pHF	(The threshold dose of pHF increased)	pHF

## Data Availability

Data available in a publicly accessible repository that does not issue DOIs.
